# Thermally Induced Transitions of d(G_4_T_4_G_3_) Quadruplexes Can Be Described as Kinetically Driven Processes

**DOI:** 10.3390/life12060825

**Published:** 2022-06-01

**Authors:** Iztok Prislan, Tomaz Urbic, Natasa Poklar Ulrih

**Affiliations:** 1 Biotechnical Faculty, University of Ljubljana, Jamnikarjeva 101, 1000 Ljubljana, Slovenia; 2 Faculty of Chemistry and Chemical Technology, University of Ljubljana, Vecna Pot 113, 1000 Ljubljana, Slovenia; tomaz.urbic@fkkt.uni-lj.si

**Keywords:** G-quadruplexes, potassium, calorimetry, polymorphism, kinetics, folding, kinetic models, fitting

## Abstract

DNA sequences that are rich in guanines and can form four-stranded structures are called G-quadruplexes. Due to the growing evidence that they may play an important role in several key biological processes, the G-quadruplexes have captured the interest of several researchers. G-quadruplexes may form in the presence of different metal cations as polymorphic structures formed in kinetically governed processes. Here we investigate a complex polymorphism of d(G_4_T_4_G_3_) quadruplexes at different K^+^ concentrations. We show that population size of different d(G_4_T_4_G_3_) quadruplex conformations can be manipulated by cooling rate and/or K^+^ concentration. We use a kinetic model to describe data obtained from DSC, CD and UV spectroscopy and PAGE experiments. Our model is able to describe the observed thermally induced conformational transitions of d(G_4_T_4_G_3_) quadruplexes at different K^+^ concentrations.

## 1. Introduction

Understanding the relationship between the structure of biological macromolecules and the thermodynamic or kinetic properties that dictate stability and binding with other molecules remains one of the most important problems in biochemistry and biotechnology [1]. Such knowledge is crucial to understanding biological processes and to design more efficient pharmaceutical ligands. The laws of thermodynamics and kinetics do not tell us directly about molecular structures or mechanisms, although we can use the results of thermodynamic property measurements to help us interpret molecular mechanisms of a system [2].

Over the past couple of decades, DNA sequences that are both rich in guanines and can form four-stranded structures, called G-quadruplexes, have captured the interest of several major laboratories all over the world. This is mainly due to the growing evidence that G-quadruplexes may play an important role in several key biological processes [3,4]. Even though much progress has been made in understanding kinetic and thermodynamic factors responsible for the structural interconversion of several quadruplex structures, there are still many factors that govern the formation of G-quadruplexes and their physico-chemical properties that are poorly understood.

Four guanine bases linked by Hoogsteen type hydrogen bonds form a cyclic coplanar G-tetrads which can stack on top of another tetrad to form G-quadruplex structures [5,6]. These structures are additionally stabilized by metal cations that are selectively bound in the central cavity between the G-quartets. Cation coordination is essential for the stabilization of G-quadruplexes, and monovalent and divalent ions have been shown to influence the structure and stability of G-quadruplexes. By compiling a number of studies, one can estimate that G-quadruplex stabilization follows the general trend: Sr^2+^ > K^+^ > Ca^2+^ > NH^4+^, Na^+^, Rb^+^ > Mg^2+^ > Li^+^ ≥ Cs^+^ [7].

Changing the type or concentration of cations can induce the formation of new long-lived G-quadruplex conformations with identical nucleotide sequence. For instance, oligonucleotide d(G_2_AG_2_AG) forms a bimolecular G-quadruplex at low Na^+^ concentration and a tetramolecular G-quadruplex at higher Na^+^ concentration [8]. Furthermore, d(G_3_T_4_G_3_) forms only hairpin dimer G-quadruplex structures in the presence of Na^+^ ions, while in the presence of K^+^ ions both hairpin dimer and linear, four-stranded G-quadruplex structures were observed [9]. This phenomenon is known as G4 polymorphism [10,11,12] and can even lead to concurrent folding isomers in heterogenous ensembles [13,14,15]. The polymorphism of GQ structures is determined by strand orientation (i.e., parallel vs. antiparallel), the conformation of the glycosidic bonds, and the loop topology (e.g., lateral loops vs. diagonal loops). These variations in the structure might be due to several reasons, such as the cationic coordination, π-stacking interactions, hydrogen bonding and hydrophobic effects [16]. Different mechanisms have been proposed to describe folding/unfolding pathways of G-quadruplexes. Some of them are sequential and do not consider side-reactions [17] while others consist of several parallel pathways [18,19].

The behavior of G-quadruplexes in the presence of potassium ions is of particular interest due to the high intracellular concentrations. Chaires et al. have suggested, based on NMR, fluorescence, CD experiments, and molecular modeling, that model telomeric human DNA, 5′-AGGGTTAGGGTTAGGGTTAGGG-3′ (Tel22) in K^+^ solutions appears as an equilibrium mixture of two (3 + 1) hybrid-type G-quadruplex structures, Hybrid-1 and Hybrid-2 [20]. This was later confirmed by Bončina et al., who successfully described the unfolding/folding mechanism of Tel22 and showed that each conformational transition depends on K^+^ concentration [11]. Grün et al. were studying the cMYC gene-promoter region and used a kinetic model to describe a novel, noncanonical folding isomerism of G-quadruplexes with more than four G-rich tracts [21]. Grey et al. utilized a variety of spectroscopies to follow the kinetics of human telomeric DNA sequences folding and unfolding, and identified previously unreported slow kinetic steps [17]. Kinetics of potassium binding to telomeric (hybrid) and c-myc (parallel) G-quadruplexes have been investigated with electrospray mass spectrometry where authors have reported that misfolded states can linger for several minutes [18]. Different folding timescales of DNA and RNA G-quadruplexes have been reported by Zhang and Balasubramanian, who discussed their findings in relation to a biological timescale [22]. The folding rate of quadruplexes and folding timescale were also investigated by Nguyen et al., who showed that a stem loop accelerates the folding of quadruplexes significantly as compared to a non-structured loop [23]. One can conclude that the formation of G-quadruplexes takes place through different mechanisms on various timescales, making the folding rate of a G-quadruplex and the effect of the environment and loop properties very important parameters to investigate.

Our laboratory has used kinetic models to describe the polymorphism of d(G_4_T_4_G_3_) quadruplexes in Na^+^ and K^+^ solutions [24,25]. To test whether this approach can be used under different environmental conditions, we followed in this work the thermally induced folding/unfolding transitions d(G_4_T_4_G_3_) quadruplexes at different K^+^ concentrations, and tried to interpret them in terms of a kinetically governed coexistence of several folded structures and their unfolded forms. We used a variety of experimental techniques (UV and CD spectroscopy, DSC, gel electrophoresis) in order to improve the significance of the proposed model and calculated parameters. We demonstrate that the d(G_4_T_4_G_3_) sequence can fold into different quadruplex structures and that the mechanism of thermally induced folding/unfolding depends on the cooling/heating rate and potassium concentration.

## 2. Materials and Methods

### 2.1. Sample Preparation and UV Spectroscopy

The d(G_4_T_4_G_3_) oligonucleotide was obtained HPLC pure from Invitrogen Co., (Carlsbad, CA, USA) and Midland Co. (Midland, CO, USA)., and used without further purification. Its concentrations in buffer solutions were determined at 25 °C spectrophotometrically using for the extinction coefficient of its single stranded form at 25 °C the value of *ε*_260_ = 105,100 M^−1^cm^−1^ estimated from the nearest-neighbor data of Cantor et al. [26]. The quadruplex concentration used in this study were 0.82, 0.60 and 0.52 mM in single strands in 25, 35 and 50 mM K^+^ solutions, respectively. The buffer used in all experiments consisted of 10 mM K-cacodylic buffer, 1 mM EDTA and different concentrations of KCl (15, 25 and 45 mM) at pH = 6.9. The starting samples were first unfolded into single-stranded form by heating in an outer thermostat at 95 °C for 5 min, cooled down to 4 °C at the cooling rates of 0.05 or 1.0 °C/min to form quadruplex structures and then used in the UV, CD, DSC and PAGE experiments.

### 2.2. CD Spectroscopy Melting Experiments

CD spectra of d(G_4_T_4_G_3_) quadruplexes in K^+^ solutions were measured as a function of temperature in an Jasco J-1500 CD Spectropolarimeter. Ellipticity, *Θ*, was measured between 5 and 95 °C in the temperature intervals of 3 °C at the average heating rate of 0.5 °C/min. CD spectra of samples (0.82, 0.60 and 0.52 mM in single strands) prepared at cooling rates of 0.05 or 1.0 °C/min, corrected for the corresponding buffer contribution, were collected between 215 and 320 nm in a 0.25 mm cuvette at 60 nm/min, with a signal averaging time of 2 s and a 5 nm bandwidth. Melting curves were obtained by plotting ellipticity at *λ* = 290 nm vs. temperature.

### 2.3. Gel Electrophoresis

G-quadruplex structures formed upon the cooling of single-stranded DNA at different rates of 0.05 or 1.0 °C/min and at different potassium concentrations were studied by non-denaturing PAGE performed on 20% polyacrylamide gels supplemented with 25, 35 and 55 mM KCl. G-quadruplex samples were loaded on gels and the electrophoreses were run at 5 °C (5 h), 20 °C (3.5 h) and 35 °C (1.7 h), all at 10 V/cm (*I* = 300 mA). Bands in the gels were followed by UV shadowing at *λ* = 254 nm. To facilitate comparisons between the bands observed with different samples, the single-stranded d(5′-AGAAGAAAAGA-3′) and d(5′-TCTTTTCTTCT-3′) and double-stranded (5′-AGAAGAAAAGA-3 mixed with 5′-TCTTTTCTTCT-3′) 11-mere control oligonucleotides were used.

After UV shadowing, experiment gels were submerged in ethidium bromide solution and bands were recorded in fluorescent mode at an excitation wavelength of 290 nm.

### 2.4. Differential Scanning Calorimetry (DSC)

DSC experiments were performed using the Nano DSC III instrument (Calorimetry Sciences Corp., Lindon, UT, USA) and the Nano DSC instrument (TA Instruments, New Castle, DE, USA) on samples prepared by cooling in an outer thermostat. Samples were first heated to 95 °C and cooled down to four °C at the selected rate (0.05 or 1.0 °C/min). Samples were then loaded to DSC and the first DSC melting scans were recorded at the selected heating rate (0.5, 1.0 and 2.0 °C/min). Next, an annealing scan was recorded at a cooling rate of 1.0 °C, followed by another melting scan at the selected heating rate. The corresponding baseline (buffer-buffer) scans were subtracted from the unfolding/folding scans prior to their normalization and analysis. The total enthalpy of unfolding or folding, $\Delta H_{\mathrm{tot}}$, was obtained from the measured DSC thermograms as the area under the $\Delta c_{p}={\bar{c}}_{p,2}-{\bar{c}}_{p,S}$ versus *T* curve, where $\Delta c_{p}$ is the measured heat capacity ${\bar{c}}_{p,2}$ corrected for the baseline and normalized to 1 mole of quadruplex in single strands, and ${\bar{c}}_{p,S}$ is the corresponding partial molar heat capacity of the unfolded single stranded state extrapolated from high temperatures over the whole measured temperature interval. For samples prepared either at the cooling rate of 0.05 or 1.0 °C/min, the measured DSC heating and cooling curves were highly reproducible at all measured heating and cooling rates above ~10 °C.

### 2.5. Model Analysis of Structural Transitions

**Figure 1:**
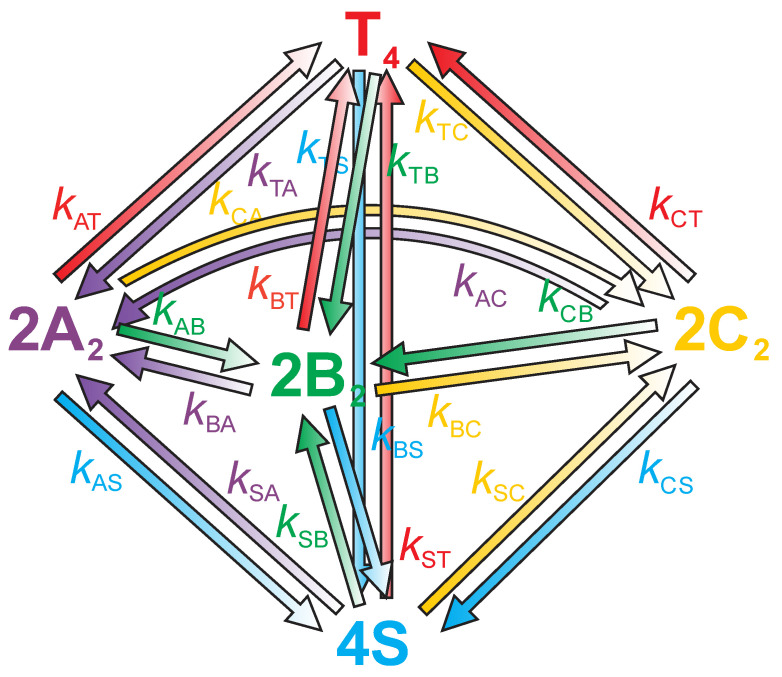
Proposed kinetic model for the thermally induced folding/unfolding transitions of d(G_4_T_4_G_3_) in K^+^ solutions which assumes the coexistence and interconversions of one tetramolecular quadruplex T_4_, three bimolecular quadruplexes A_2_, B_2_ and C_2_ and the unfolded single strands S. *k*_ij_ are rate constants for each i→j transition step.

We attempted to interpret the observed DSC data in terms of the already described complex model (Figure 1) [24], which considers the thermally induced folding/unfolding transitions of d(G_4_T_4_G_3_) quadruplexes in the presence of K^+^ ions as a global kinetic transition process that involves one parallel tetramolecular quadruplex structure (T_4_), three bimolecular quadruplexes that possibly exhibit characteristics of parallel, anti-parallel and/or hybrid structures (A_2_, B_2_ and C_2_), and the corresponding single strands (S).

In order to describe the DSC data, we used the previously reported model function [24]:

(1) $$\Delta c_{p}={\bar{c}}_{p,2}-{\bar{c}}_{p,S}=-\frac{d\alpha_{A}}{dT}\Delta H_{A}-\frac{d\alpha_{B}}{dT}\Delta H_{B}-\frac{d\alpha_{C}}{dT}\Delta H_{C}-\frac{d\alpha_{T}}{dT}\Delta H_{T}$$

in which at any *T* in the measured temperature interval ${\bar{c}}_{p,2}$ is the measured partial molar heat capacity of the solute (DNA) expressed per moles of single strands, ${\bar{c}}_{p,S}$ is the corresponding measured heat capacity of the unfolded quadruplex form occurring at high temperatures extrapolated to *T*, and $\alpha_{A}$, $\alpha_{B}$, $\alpha_{C}$ and $\alpha_{T}$ are the corresponding fractions of quadruplex species present in the solution. The $\frac{d\alpha }{dT}$ terms needed to calculate $\Delta c_{p}$ are obtained by taking into account the rates of reactions predicted by the model and the heating or cooling rate, $r=\frac{dT}{dt}$, at which the DSC experiment is performed. The rates of reactions can be expressed using the Arrhenius relation $k_{ij}=A_{ij}e^{\left(-E_{ij}/RT\right)}=e^{A_{ij}^{\prime }}e^{\left(-E_{ij}/RT\right)}=e^{\left(A_{ij}^{\prime }-E_{ij}/RT\right)}$, where $k_{ij}$ represents rate constant, $A_{ij}=e^{A_{ij}^{\prime }}$ frequency factor and $E_{ij}$ activation energy for corresponding ij step. Unit of $e^{A_{ij}^{\prime }}$ is s^−1^ for unfolding and s^−1^ M^−1^ for folding transitions of A_2_, B_2_ and C_2_ (s^−1^ M^−3^ for folding transition of T_4_ from S, i.e. $e^{A_{\mathrm{ST}}^{\prime }}$) and unit of $E_{ij}$ is cal/mol.

For a given total concentration of DNA in the single-stranded form,$\;c_{\mathrm{tot}}$ (in unit mol/L), expressed as $c_{\mathrm{tot}}=c_{S}+2c_{A}+2c_{B}+2c_{C}+4c_{T}$, the dα/dT terms needed to calculate $\Delta c_{p}$ are obtained by taking into account the rates of reactions predicted by the model (Figure 1):

$$\begin{array}{c} -\frac{d\alpha_{A}}{dT}=\frac{1}{r}[\left(k_{\mathrm{AB}}+k_{\mathrm{AS}}+k_{\mathrm{AC}}\right)\alpha_{A}+C_{\mathrm{tot}}k_{\mathrm{AT}}\alpha_{A}^{2}-k_{\mathrm{BA}}\alpha_{B}-k_{\mathrm{TA}}\alpha_{T}-k_{\mathrm{CA}}\alpha_{C}\\ -2k_{\mathrm{SA}}C_{\mathrm{tot}}{\left(1-\alpha_{A}-\alpha_{B}-\alpha_{C}-\alpha_{T}\right)}^{2}] \end{array}$$

$$\begin{array}{c} -\frac{d\alpha_{B}}{dT}=\frac{1}{r}[\left(k_{\mathrm{BS}}+k_{\mathrm{BC}}+k_{\mathrm{BA}}\right)\alpha_{B}+C_{\mathrm{tot}}k_{\mathrm{BT}}\alpha_{B}^{2}-k_{\mathrm{AB}}\alpha_{A}-k_{\mathrm{TB}}\alpha_{T}-k_{\mathrm{CB}}\alpha_{C}\\ -2k_{\mathrm{SB}}C_{\mathrm{tot}}{\left(1-\alpha_{A}-\alpha_{B}-\alpha_{C}-\alpha_{T}\right)}^{2}] \end{array}$$

$$\begin{array}{c} -\frac{d\alpha_{C}}{dT}=\frac{1}{r}[\left(k_{\mathrm{CB}}+k_{\mathrm{CS}}+k_{\mathrm{CA}}\right)\alpha_{C}+C_{\mathrm{tot}}k_{\mathrm{CT}}\alpha_{C}^{2}-k_{\mathrm{BC}}\alpha_{B}-k_{\mathrm{TC}}\alpha_{T}-k_{\mathrm{AC}}\alpha_{A}\\ -2k_{\mathrm{SC}}C_{\mathrm{tot}}{\left(1-\alpha_{A}-\alpha_{B}-\alpha_{C}-\alpha_{T}\right)}^{2}] \end{array}$$

$$\begin{array}{c} -\frac{d\alpha_{T}}{dT}=\frac{1}{r}[\left(k_{\mathrm{TA}}+k_{\mathrm{TB}}+k_{\mathrm{TC}}+k_{\mathrm{TS}}\right)\alpha_{T}-C_{\mathrm{tot}}k_{\mathrm{AT}}\alpha_{A}^{2}-C_{\mathrm{tot}}k_{\mathrm{BT}}\alpha_{B}^{2}-C_{\mathrm{tot}}k_{\mathrm{CT}}\alpha_{C}^{2}\\ -4k_{\mathrm{ST}}C_{\mathrm{tot}}^{3}{\left(1-\alpha_{A}-\alpha_{B}-\alpha_{C}-\alpha_{T}\right)}^{4}] \end{array}$$

When fitting the kinetic model (Figure 1) to our experimental data, all parameters were allowed to be used. We found out that not all of them were necessary to obtain an acceptable fit of model functions to experimental data. We neglected these parameters (set $k_{\mathrm{ij}}$ to 0), thus simplifying the system of equations and increasing the significance of the remaining parameters:

$$-\frac{d\alpha_{A}}{dT}=\frac{1}{r}\left[\left(k_{\mathrm{AB}}+k_{\mathrm{AS}}\right)\alpha_{A}-k_{\mathrm{BA}}\alpha_{B}\right]$$

$$-\frac{d\alpha_{B}}{dT}=\frac{1}{r}\left[\left(k_{\mathrm{BS}}+k_{\mathrm{BC}}+k_{\mathrm{BA}}\right)\alpha_{B}+C_{\mathrm{tot}}k_{\mathrm{BT}}\alpha_{B}^{2}-k_{\mathrm{AB}}\alpha_{A}-2k_{\mathrm{SB}}C_{\mathrm{tot}}{\left(1-\alpha_{A}-\alpha_{B}-\alpha_{C}-\alpha_{T}\right)}^{2}\right]$$

$$-\frac{d\alpha_{C}}{dT}=\frac{1}{r}\left[k_{\mathrm{CS}}\alpha_{C}-k_{\mathrm{BC}}\alpha_{B}\right]$$

$$-\frac{d\alpha_{T}}{dT}=\frac{1}{r}\left[k_{\mathrm{TS}}\alpha_{T}-C_{\mathrm{tot}}k_{\mathrm{BT}}\alpha_{B}^{2}\right]$$

To solve this system of differential equations for a given set of adjustable parameters at each measured heating and cooling rate, the Cash-Karp adaptive step-size controlled Runge-Kutta method was employed [27], and the obtained solutions ($\alpha_{i}$ and $\frac{d\alpha_{i}}{dT}$) were used to calculate the corresponding model function. The calculation started at a high temperature and a set cooling rate. The initial composition was $\alpha_{S}=1$. When the low temperature was reached, the obtained composition was a starting point for heating. The “best fit” adjustable parameters were calculated from global fitting model functions to the experimental DSC curves using the non-linear minimization of the corresponding $\chi^{2}$ function. The values of adjustable parameters at the global minimum of $\chi^{2}$ are considered to be the best descriptors of the experimental $\Delta c_{p}$ vs. *T* curve, and therefore are used to characterize the kinetics and thermodynamics for all steps in the suggested model mechanism.

## 3. Results and Discussion

### 3.1. Observing Polymorphism of d(G_4_T_4_G_3_) Quadruplexes in K^+^ Solution

**Figure 2:**
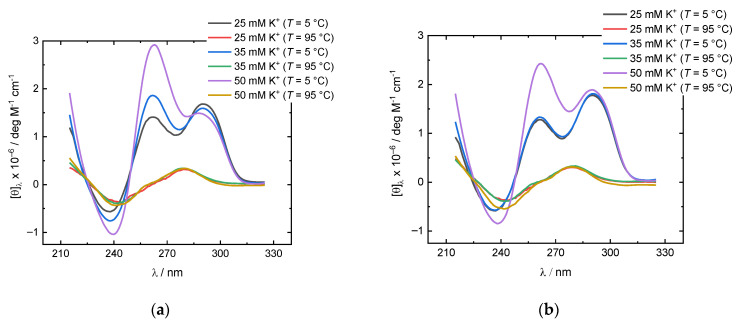
Temperature dependence of CD spectra of d(G_4_T_4_G_3_) quadruplexes in K^+^ solutions (25, 35 and 50 mM): (**a**) d(G_4_T_4_G_3_) quadruplexes prepared at the cooling rate of 0.05 °C/min; (**b**) d(G_4_T_4_G_3_) quadruplexes prepared at the cooling rate of 1 °C/min. The measured ellipticity was normalized to 1 M single strand concentration and 1 cm light path length.

We measured the CD spectra of d(G_4_T_4_G_3_) samples prepared in 25, 35 and 50 mM K^+^ solutions at the cooling rate of 0.05 °C/min (Figure 2a), and at the cooling rate of 1.0 °C/min (Figure 2b). The CD spectra is often used to interpret the structure of G-quadruplexes. CD-spectra with positive peak at ~290 nm and negative peak at ~263 nm are characteristic of the anti-parallel type quadruplex structures. On the other hand, the parallel type structures exhibit CD spectra with a positive peak at ~263 nm and negative peak at ~240 nm [28,29]. Hybrid quadruplex structures may also be involved in thermally induced folding/unfolding transitions [11,30,31]. The comparison of three spectra in Figure 2a shows that the peak at 263 nm increases, and the peak at 290 nm decreases with increasing K^+^ concentration. The same behavior can be observed when samples are prepared at the cooling rate of 1.0 °C/min (Figure 2b). Thus, we can conclude that increasing K^+^ concentration favors the formation of a structure with spectral properties similar to parallel type quadruplexes. Interestingly, when comparing the CD spectra of samples prepared at different cooling rates, we can observe that a slower cooling rate results in increased peak at 263 nm and a decreased peak at 290 nm. These results show that besides changing K^+^ concentration, different cooling rates can be used to manipulate the structure of d(G_4_T_4_G_3_) quadruplexes during thermally induced folding. The cooling rate dependence of structural properties of d(G_4_T_4_G_3_) quadruplexes point to the conclusion that thermally induced folding/unfolding transitions of d(G_4_T_4_G_3_) quadruplexes are a kinetically driven process. Furthermore, because of the increased peak at 263 nm after a slow cooling rate, we can assume that a slower cooling rate also favors the formation of a structure with spectral properties similar to parallel-type quadruplexes.

**Figure 3:**
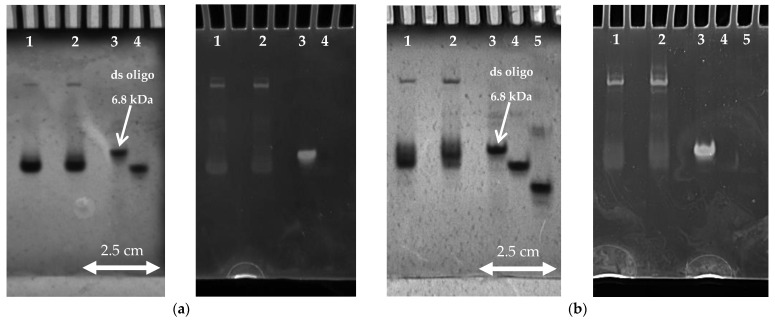
Non denaturing PAGE (20%) of d(G_4_T_4_G_3_) quadruplexes prepared at the cooling rate of either 1 °C/min (1st lane) or 0.05 °C/min (2nd lane) and followed by UV shadowing (*λ* = 254 nm) and Fluorescence (*λ* = 590 nm): (**a**) d(G_4_T_4_G_3_) quadruplexes prepared in 25 mM K^+^ solution and measured at a constant temperature of 5 °C. As a control oligonucleotide double stranded d(5′-AGAAGAAAAGA-3′; 5′-TCTTTTCTTCT-3′) (3rd lane) and 11-mere single stranded d(5′-TCTTTTCTTCT-3′) (4th lane) markers were used;): (**b**) d(G_4_T_4_G_3_) quadruplexes prepared in 50 mM K^+^ solution and measured at a constant temperature of 5 °C. As control oligonucleotide, double stranded d(5′-AGAAGAAAAGA-3′; 5′-TCTTTTCTTCT-3′) (3rd lane), single stranded d(5′-TCTTTTCTTCT-3′) (4th lane) and single stranded d(5′-AGAAGAAAAGA-3′) (5th lane) markers were used.

The CD spectra suggest that at least two structures with antiparallel and parallel spectral properties (for reasons of clarity we will call them “antiparallel” and “parallel” structures) coexist in solution after cooling, and that the ratio between antiparallel and parallel structures depends on the potassium concentration and a cooling rate. To confirm these results, we performed PAGE experiments where slow and fast cooled d(G_4_T_4_G_3_) samples in 25 mM and 50 mM K^+^ solutions were run on the gels at 5 °C, the same temperature at which CD-spectra were recorded (Figure 3). Control oligonucleotide markers (single-stranded d(5′-TCTTTTCTTCT-3′), single-stranded d(5′-AGAAGAAAAGA-3′) and double-stranded (5′-AGAAGAAAAGA-3′ mixed with 5′-TCTTTTCTTCT-3′)) were used to ascribe the size of the molecules to the positions of the bands, and to compare different gels. The gel experiments show that all samples exhibit at least three bands where two fast migrating bands are very close to each other, indicating the presence of at least two structures similar in size and charge. Furthermore, the samples prepared at the cooling rate of 0.05 °C/min (2nd lane) exhibit a more pronounced band, positioned just behind the fastest migrating band, which in accordance with CD-spectra probably represents a parallel structure. This observation can be further confirmed by comparing gels at different K^+^ concentrations since the slower of the two fast migrating bands is more pronounced at 50 mM K^+^ concentration. Besides the two fast migrating bands, one slow migrating band can be observed at both K^+^ concentrations and cooling rates, but the intensity is less pronounced in samples prepared at cooling rate of 1.0 °C/min (1st lane). Interestingly, staining the gels with ethidium bromide results in high intensity fluorescence signals for the slowest migration band and double stranded marker, whereas very weak signals can be observed for the fastest migrating bands. Based on the fluorescence intensity, we can conclude that ethidium bromide interacts with the structure in the slowest migrating band in a similar way to the double stranded marker, and that interaction with quadruplexes is weaker compared to duplex DNA as already observed before [32]. The position and difference in fluorescence intensity between fast and slow migrating bands indicates the significant differences of complexes, and we believe that the slower migrating band corresponds to some highly ordered structure, for example the parallel tetramolecular d(G_4_T_4_G_3_)_4_ quadruplex.

### 3.2. Thermally Induced Structural Transitions and Model Analysis

**Figure 4:**
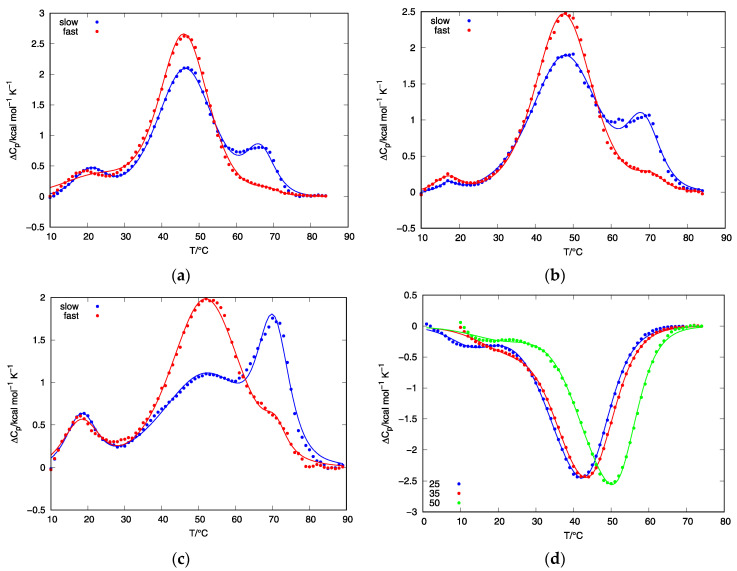
Fitting the model function to the heating and cooling DSC thermograms of d(G_4_T_4_G_3_) quadruplexes in the presence of 25, 35 and 50 mM K^+^ ions: (**a**) The Δ*c*_p_ vs. *T* curves measured at the heating rate of 1 °C/min in the presence of 25 mM K^+^ ions for samples prepared by controlled cooling in an outer thermostat at the rate of 0.05 °C/min (red line) or 1.0 °C/min (blue line); (**b**) The Δ*c*_p_ vs. *T* curves measured at the heating rate of 1 °C/min in the presence of 35 mM K^+^ ions for samples prepared by controlled cooling in an outer thermostat at the rate of 0.05 °C/min (red line) or 1.0 °C/min (blue line); (**c**) The Δ*c*_p_ vs. *T* curves measured at the heating rate of 1 °C/min in the presence of 50 mM K^+^ ions for samples prepared by controlled cooling in an outer thermostat at the rate of 0.05 °C/min (red line) or 1.0 °C/min (blue line); (**d**) The Δ*c*_p_ vs. *T* curves measured at the cooling rate of 1 °C/min in the presence of 25 (blue line), 35 (red line) and 50 (green line) mM K^+^ ions. In all panels full lines represent model–based Δ*c_p_* vs. *T* curves calculated from (1).

To observe the thermal behavior of d(G_4_T_4_G_3_) starting samples, we have conducted a series of DSC and CD experiments. DSC heating thermograms of d(G_4_T_4_G_3_) samples measured in 25, 35, and 50 mM K^+^ solutions and prepared at the cooling rate of either 0.05 or 1.0 °C/min exhibit at least three peaks suggesting that the melting processes consist of several conformational transitions (Figure 4). Samples prepared at the cooling rate of 0.05 °C/min exhibit peaks at ~20 °C, ~45 °C and ~65 °C, suggesting the coexistence of at least three quadruplex structures, which is in line with PAGE experiments. Increasing the K^+^ concentration decreases the intensity of the second peak and increases the intensity of the third. Together with the data from CD experiments, this suggests that the third peak corresponds to the unfolding of a parallel structure and that the parallel structure is thermally more stable than the antiparallel. This can be further proved by PAGE experiments of samples prepared at a slow cooling rate at 50 mM K^+^ concentration (Figure 3b) where the increased intensity of the slower of the two fast migrating bands can be observed. Samples prepared at the cooling rate of 1.0 °C/min also exhibit three peaks, but the third peak is not as expressed as in the case of a slower cooling rate. These results, together with the CD (Figure 2) and PAGE (Figure 3) experiments, strongly suggest that the thermal unfolding transition of d(G_4_T_4_G_3_) in the presence of K^+^ ions may be considered as a combination of several kinetically governed steps.

The total enthalpy of unfolding, $\Delta H_{\mathrm{tot}}$, was calculated from the experimentally obtained DSC thermograms (total area under the Δ$C_{P}$ versus *T* curve). Analysis of the total area under measured heating DSC thermograms resulted in very similar overall enthalpies of unfolding, Δ*H*_tot_ ≈ 56 kcal/mol of double stranded quadruplex, regardless of the cooling rate or K^+^ concentration used. From these Δ*H*_tot_ values, the enthalpy of quadruplex unfolding is estimated to be about 19 kcal/mol of G-quartets, which agrees well with the literature values reported for G-quartet formation in the presence of K^+^ ions [33,34,35]. After the first melting scan was recorded at the selected heating rate (0.5, 1.0 and 2.0 °C/min), the sample was left in DSC and a cooling scan was recorded at the rate of 1.0 °C/min, followed by another melting scan at the selected heating rate. Almost no differences were observed when comparing the second melting scans to the melting scan at corresponding heating rates obtained from samples prepared in an outer thermostat at the cooling rate of 1 °C/min. This confirms the kinetic nature of the folding/unfolding transition of d(G_4_T_4_G_3_) in the presence of K^+^ ions.

Interestingly, DSC thermograms show transition at low temperatures. Even though the reproducibility and reliability of the measured DSC peaks between 4 °C and ~20 °C is rather poor, PAGE experiments confirmed that there is an additional structure present at low temperatures that disappears at temperatures over 20 °C (Figure S2). The position and difference in fluorescence intensity between fast and slow migrating bands (Figure 3) indicates significant differences in structures of complexes, and we believe that a slower migrating band corresponds to some highly ordered structure, for example, the parallel tetramolecular d(G_4_T_4_G_3_)_4_ quadruplex.

**Figure 5:**
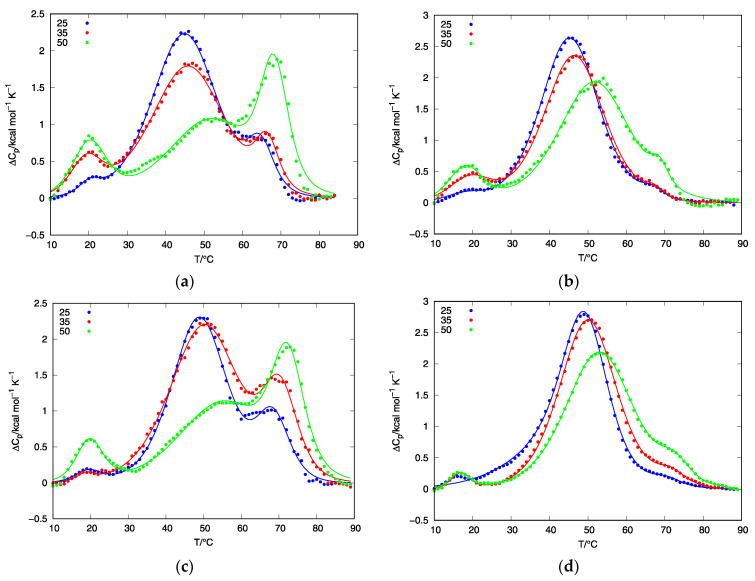
Fitting the model function to heating DSC thermograms of d(G_4_T_4_G_3_) quadruplexes at different rates in the presence of 25 (blue color), 35 (red color) and 50 (green color) mM K^+^ ions: (**a**) at the heating rate of 0.5 °C/min for samples prepared at the cooling rate of 0.05 °C/min; (**b**) at the heating rate of 0.5 °C/min for samples prepared at the cooling rate of 1.0 °C/min; (**c**) at the heating rate of 2.0 °C/min for samples prepared at the cooling rate of 0.05 °C/min; (**d**) at the heating rate of 2.0 °C/min for samples prepared at the cooling rate of 1.0 °C/min. In all panels full lines represent the model—based curves calculated from (1).

The results of DSC, PAGE and CD experiments performed on d(G_4_T_4_G_3_) quadruplexes in K^+^ solutions reveal that their thermally induced folding/unfolding transitions are kinetically governed and include the participation of at least three structures. A model function (1) was used to describe thermally induced folding/unfolding transitions of d(G_4_T_4_G_3_) quadruplexes in the presence of K^+^ ions at different concentrations. Figure 4 shows the best fit of model function to the DSC data. To further test the validity of our model, we have collected and fitted data at several heating rates (0.5, 1.0 and 2.0 °C/min) for DSC experiments. By increasing the heating rate, we can observe shifting of the DSC peaks to higher temperatures (Figure 5), which is another indication that conformational transitions of d(G_4_T_4_G_3_) in the presence of K^+^ ions are kinetically driven processes. The model function (1) with the same set of parameters (Table 1) was used to describe experimental data at all heating rates at selected K^+^ concentration.

**Figure 6:**
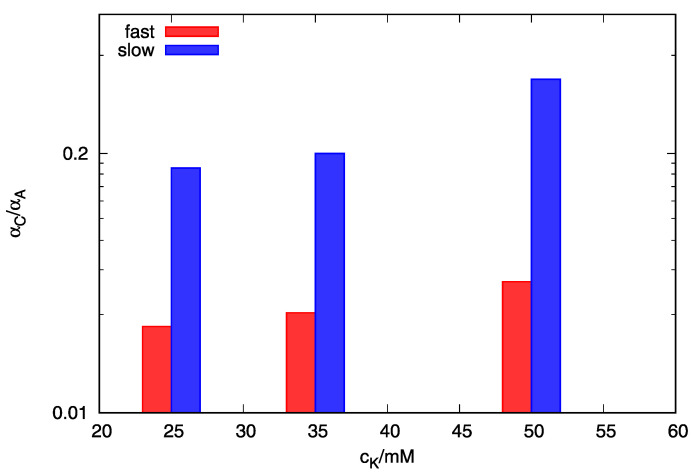
Ratio between calculated fractions of bimolecular quadruplex species C_2_ and A_2_ at different K^+^ concentrations prepared at cooling rates of 0.05 and 1.0 °C/min on a logarithm scale.

By comparing the parameters in Table 1 at different potassium concentrations, we can observe an increase of parameter *A*′_BC_, which is connected to the frequency factor for the transition from structure B_2_ to structure C_2_ by equation $A_{\mathrm{BC}}=e^{A_{\mathrm{BC}}^{\prime }}$. Also, we can observe a decrease of parameter *A*′_CS_, which is connected to frequency factor for transition from structure B_2_ to structure C_2_ by equation $A_{\mathrm{BC}}=e^{A_{\mathrm{BC}}^{\prime }}$. Frequency factors are proportional to the rate constant, *k*_ij_, which means that increase in potassium concentration will increase the population of structure C_2_. This behavior of model-based parameters correlates nicely with the experimental data which show higher populations of parallel quadruplex at *C*_K+_ = 50 mM after slow and fast rates of cooling. Calculated population distributions of bimolecular structures A_2_ and C_2_ at slower and at higher cooling rates at different potassium concentrations are shown in Figure 6. By comparing the data from Figure 4, Figure 5 and Figure 6 we can see that increase of the third peak in DSC thermograms correlates to the increase of the ratio between the calculated fractions of bimolecular quadruplex species C_2_ and A_2_. This means that the position of the third peak in DSC heating thermograms, which increases in intensity when slow cooling or high concentrations of potassium ions are used, corresponds to the unfolding of structure C_2_.

The correlation between fitting parameters was calculated for all potassium concentrations (Tables S1–S3). Most of the parameters show weak correlation but stronger correlation is observed between parameters *A*′_ij_ and *E*_ij_. Since the choice of baseline is the largest source of errors accompanying the DSC measurements, the errors were estimated by changing the baseline and plotting “top and bottom” DSC curves which served as confidence limits for our experiment (Figure S1). Applying the calculated errors simultaneously to highly correlated parameters resulted in poorer agreement between the model function and the experiment (data not shown). This suggests that within the estimated error margins the observed higher correlation between parameters *A*′_ij_ and *E*_ij_ has no significant effect on their reliability.

Fitting the model function to experimental DSC data yields temperature dependence of population of predicted quadruplex structures involved in the measured folding/unfolding process. In order to successfully interpret the observed DSC data, we had to include one additional bimolecular quadruplex structure B_2_ in our model (Figure 1). The calculated population of quadruplex structure B_2_ is negligibly small at all temperatures, suggesting that B_2_ acts as an unstable intermediate. Even though the population of B_2_ is negligibly small, its presence in our model is necessary to fit the entire cycle of DSC curves at selected potassium concentration (two different cooling rates and three different heating rates) with a single set of parameters. Efforts were made to fit the experimental data without quadruplex structure B_2_, but this less complex model was only able to satisfactorily describe either the melting or cooling curves.

**Figure 7:**
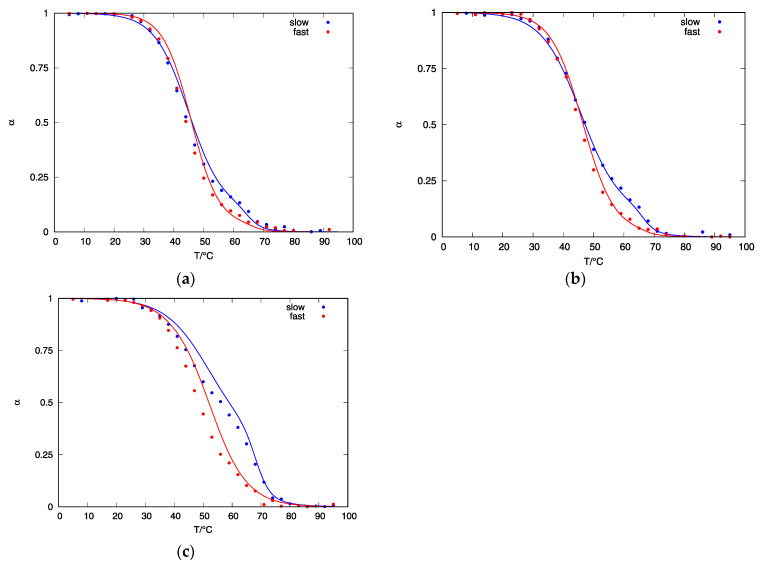
Normalized CD melting curves at λ = 290 nm of d(G_4_T_4_G_3_) quadruplexes obtained from experiments (dots) and calculated from fractions of quadruplex species present in the solution ($\alpha_{A}$, $\alpha_{B}$, $\alpha_{C}$ and $\alpha_{T}$ ) (full line) after slow (blue color) and fast (red color) cooling: (**a**) in the presence of 25 mM K^+^ ions; (**b**) in the presence of 35 mM K^+^ ions; (**c**) in the presence of 50 mM K^+^ ions.

The calculated populations of other predicted structures can be used to construct a CD-melting curve and compare it to the experimental CD-curve. Figure 7 shows normalized CD melting curves of d(G_4_T_4_G_3_) quadruplexes obtained from experiments and the CD-melting curves calculated from the concentrations of predicted structures in the solution. Good agreement between the calculated and experimental CD-melting curves can be observed at 25 and 35 mM K^+^ concentrations (Figure 7a,b). However, the agreement of model melting curve with the experimental melting curve at 50 mM K^+^ concentration is not so good but still acceptable (Figure 7c). We attribute this discrepancy to the error in estimated heating rate during CD-measurements.

Even though our proposed model is able to satisfactory describe experimental data, it is worth mentioning that the rate of quadruplex transitions can be slower than the DSC timescale [21,36]. Our DSC experiments were performed at different scanning rates, but other than that, we did not consider this issue in experimental setup and kinetic modeling. For example, if the transition between structures A_2_ and C_2_ is very slow, DSC would be unable to record it. Although the proposed kinetic model (Figure 1) allowed multiple pathways, only a minimum number of parameters was used to obtain a good agreement between model prediction and experimental results. The elimination of parameters might be interpreted as the nonexistence of corresponding transitions whereas in reality, the transitions were too slow to be recorded by DSC in dynamic mode or the change in enthalpy was close to zero. Additional experiments should be performed to quantify the real number of species in mixture and to define which (if any) are kinetically trapped intermediates. Fully matched quadruplex assemblies are the most stable structures but mismatched species can form at a faster rate and have long lifetimes [37,38,39]. For example, if we use data from Table 1 at $C_{K^{+}}=25\;\mathrm{mM}\;$ and *T* = 281 K, we can calculate the formation and dissociation rates of structures A_2_ ($k_{\mathrm{BA}}=19.6\;s^{-1}$, $k_{\mathrm{AS}}=2.6\cdot \Delta 10^{-4}\;s^{-1}$) and C_2_ ($k_{\mathrm{BC}}=0.05\;s^{-1}$, $k_{\mathrm{CS}}=5.0\cdot \Delta 10^{-6}\;s^{-1}$). Since $k_{\mathrm{BA}}>k_{\mathrm{BC}}$ and $k_{\mathrm{AS}}<k_{\mathrm{CS}}$, we may conclude that although folding of structure A_2_ is faster, C_2_ is thermodynamically more stable. When the concentration of potassium is increased ($C_{K^{+}}=50\;\mathrm{mM}$), the folding rate of A_2_ is decreased ($k_{\mathrm{BA}}=10.7\;s^{-1}$) while the folding rate of C_2_ is increased ($k_{\mathrm{BC}}=0.66\;s^{-1}$). At the same time, the increased potassium concentration further stabilizes C_2_ by decreasing the dissociation rate ($k_{\mathrm{CS}}=9.2\times 10^{-8}\;s^{-1}$). One might jump to a conclusion that A_2_ is kinetically trapped and C_2_ is a thermodynamically stable structure, but further experiments on a longer timescale are needed to confirm this hypothesis.

## 4. Conclusions

The results of DSC, PAGE and CD experiments performed on d(G_4_T_4_G_3_) quadruplexes at different K^+^ concentrations suggest a complex thermally induced folding/unfolding mechanism that includes the participation of at least three quadruplex structures. To describe the effects of cooling/heating rates and potassium concentration on thermally induced formation/disruption of d(G_4_T_4_G_3_) quadruplex structures, we developed a kinetic model that involves one parallel tetramolecular quadruplex structure, three bimolecular quadruplexes and the corresponding single strands. The model was able to describe an interesting behavior of the d(G_4_T_4_G_3_) quadruplex where the distribution of structures with different spectral and gel mobility properties could be manipulated by cooling rate and the concentration of potassium ions. We would like to emphasize that following thermally induced folding/unfolding transitions by DSC is a dynamic measurement. There could be additional transitions present in our system but if their rate is slow relative to the DSC timescale they remained undetected.

We believe that our research provides new insights into how conformational changes of highly ordered G-quadruplexes can be manipulated by the cooling rate of thermally induced folding and concentration of potassium ions. Also, we have shown how to use a kinetic model to describe the thermally induced folding/unfolding processes of a d(G_4_T_4_G_3_)_4_ quadruplex and obtain kinetic and thermodynamic parameters for individual elementary steps. It would be interesting to include the concentration of potassium ions as the fitting parameter in our model, although additional experiments would have to be performed at several other K^+^ concentrations. Furthermore, additional experiments on a longer timescale should be performed to assess whether the system is at equilibrium and not kinetically trapped. However, this remains the focus of our future work.

## Figures and Tables

**Table 1:** Calculated adjustable parameters for the kinetic model in Figure 1 which were used to describe thermally induced folding/unfolding transitions. The unit of $e^{A_{ij}^{\prime }}$ is s^−1^ for unfolding and s^−1^ M^−1^ for folding transitions of A_2_, B_2_ and C_2_ and unit of $E_{ij}$ is cal/mol. Experimentally determined enthalpies of transition, Δ*H* (cal/mol single strands) were as follows: Δ*H*_A_ = Δ*H*_B_ = Δ*H*_C_ = (3.1 ± 0.2)⋅10^4^, Δ*H*_T_ = (3.6 ± 0.2)⋅10^4^.

Parameter	*C*_K+_ = 25 mM	*C*_K+_ = 35 mM	*C*_K+_ = 50 mM
*A*′_BS_	74 ± 5	79 ± 5	81 ± 5
*A*′_SB_	19 ± 3	19 ± 3	19 ± 3
*A*′_AS_	49 ± 2	49 ± 2	49 ± 2
*A*′_TS_	100 ± 10	100 ± 10	100 ± 10
*A*′_CS_	54 ± 2	53 ± 2	50 ± 2
*A*′_BA_	15.5 ± 0.8	15.0 ± 0.8	14.9 ± 0.8
*A*′_AB_	7.2 ± 0.4	7.2 ± 0.4	7.2 ± 0.4
*A*′_BT_	66 ± 4	66 ± 4	66 ± 4
*A*′_BC_	24 ± 2	25 ± 2	30 ± 2
*E*_BS_	(3.9 ± 0.2) × 10^4^	(4.1 ± 0.2) × 10^4^	(4.0 ± 0.2) × 10^4^
*E*_AS_	(3.2 ± 0.1) × 10^4^	(3.2 ± 0.1) × 10^4^	(3.2 ± 0.1) × 10^4^
*E*_TS_	(6.0 ± 0.2) × 10^4^	(6.0 ± 0.2) × 10^4^	(6.0 ± 0.2) × 10^4^
*E*_CS_	(3.7 ± 0.1) × 10^4^	(3.6 ± 0.1) × 10^4^	(3.7 ± 0.1) × 10^4^
*E*_BA_	(7.0 ± 0.5) × 10^3^	(7.0 ± 0.5) × 10^3^	(7.0 ± 0.5) × 10^3^
*E*_BT_	(3.2 ± 0.1) × 10^4^	(3.2 ± 0.1) × 10^4^	(3.2 ± 0.1) × 10^4^
*E*_BC_	(1.5 ± 0.1) × 10^4^	(1.4 ± 0.1) × 10^4^	(1.7 ± 0.1) × 10^4^

## Data Availability

The data presented in this study are available on request from the corresponding author.

## Supplementary Materials

The following supporting information can be downloaded at: https://www.mdpi.com/article/10.3390/life12060825/s1, Figure S1. In order to estimate experimental error, two baselines were used to subtract DSC data. First baseline yielded largest overall area under thermogram (empty circles) and the second baseline yielded smallest overall area under thermogram (full circles). The Δ*c_p_* vs. T curves were measured at the heating rate of 1.0 °C/min for samples prepared at the cooling rate of 0.05 °C/min; (a) in 25 mM K^+^ solution (b) in 35 mM K^+^ solution; (c) in 50 mM K^+^ solution, Figure S2. Non denaturing PAGE (20%) of d(G_4_T_4_G_3_) quadruplexes prepared at the cooling rate of either 1.0 °C/min (1st lane) or 0.05 °C/min (2nd lane) in 50 mM K^+^ solution and followed by UV shadowing (*λ* = 254 nm) and Fluorescence (*λ* = 590 nm): (a) d(G_4_T_4_G_3_) quadruplexes measured at constant temperature of 5 °C; (b) d(G_4_T_4_G_3_) quadruplexes prepared at constant temperature of 20 °C. (c) d(G_4_T_4_G_3_) quadruplexes prepared at constant temperature of 35 °C. As control oligonucleotide double stranded d(5’-AGAAGAAAAGA-3’; 5’-TCTTTTCTTCT-3’) (3rd lane), single stranded d(5’-TCTTTTCTTCT-3’) (4th lane) and single stranded d(5’-AGAAGAAAAGA-3’) (5th lane) markers were used. Table S1: Correlation matrix corresponding to the model analysis of the DSC melting and cooling thermograms for concentration K^+^ ions equal to 25 mM, Table S2: Correlation matrix corresponding to the model analysis of the DSC melting and cooling thermograms for concentration K^+^ ions equal to 35 mM, Table S3: Correlation matrix corresponding to the model analysis of the DSC melting and cooling thermograms for concentration K+ ions equal to 50 mM.
