# Supplementary material for: Thermally Induced Transitions of d(G4T4G3) Quadruplexes Can Be Described as Kinetically Driven Processes
Source: Life (Basel). 2022 Jun 1;12(6):825. doi: 10.3390/life12060825 (PMC9225023; doi:10.3390/life12060825)
Supplement: Supplementary file 1 [file life-12-00825-s001.zip › life-1693725-supplementary.pdf]

Supplementary Material

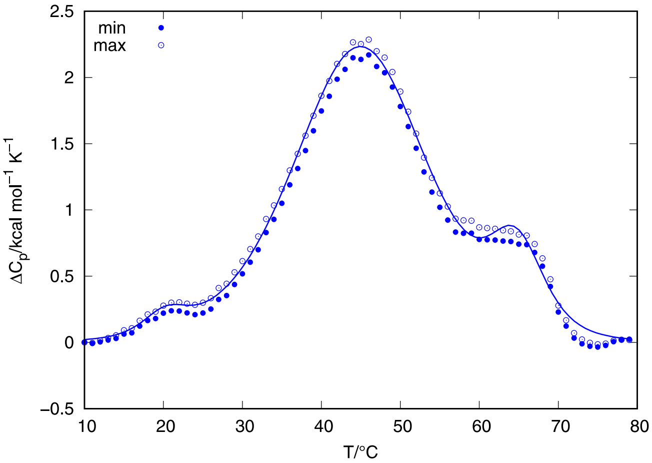

(a)

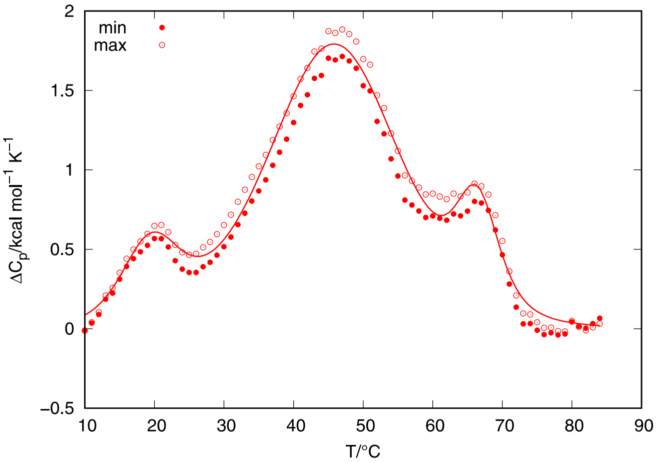

(b)

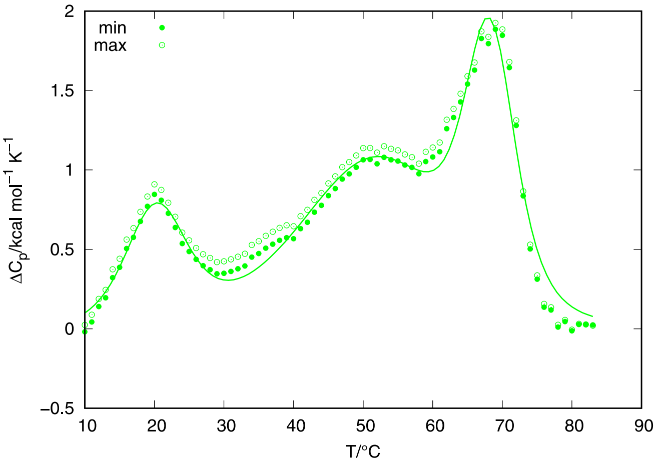

(c)

Figure S1. In order to estimate experimental error, two baselines were used to subtract DSC data. First baseline yielded largest overall area under thermogram (empty circles) and the second baseline yielded smallest overall area under thermogram (full circles). The Δc_p vs T curves were measured at the heating rate of 1.0 °C/min for samples prepared at the cooling rate of 0.05 °C/min; (**a**) in 25 mM K^+ solution (**b**) in 35 mM K^+ solution; (**c**) in 50 mM K^+ solution.

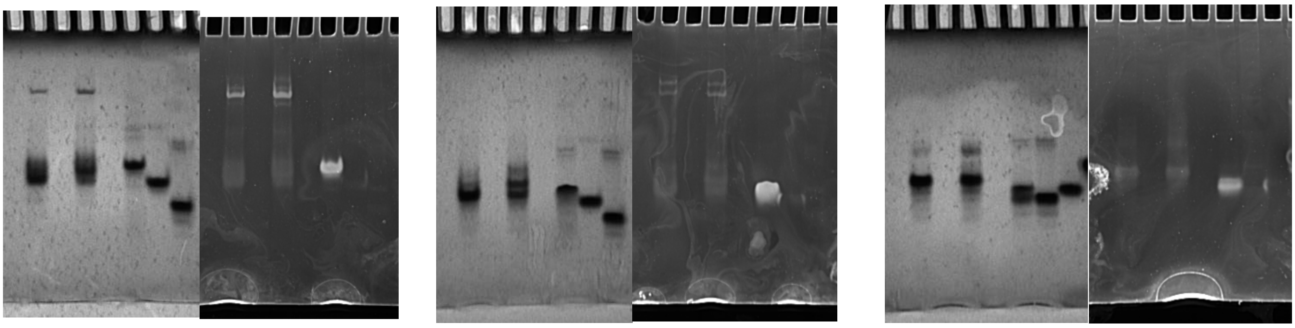

(a) (b) (c)

Figure S2. Non denaturing PAGE (20 %) of d($G_4T_4G_3$) quadruplexes prepared at the cooling rate of either 1.0 °C/min (1st lane) or 0.05 °C/min (2nd lane) in 50 mM K^+ solution and followed by UV shadowing (λ=254 nm) and Fluorescence (λ=590 nm): (**a**) d($G_4T_4G_3$) quadruplexes measured at constant temperature of 5 °C; (**b**) d($G_4T_4G_3$) quadruplexes prepared at constant temperature of 20 °C. (**c**) d($G_4T_4G_3$) quadruplexes prepared at constant temperature of 35 °C. As control oligonucleotide double stranded d(5′-AGAAGAAAAGA-3′; 5′-TCTTTTCTTCT-3′) (3rd lane), single stranded d(5′-TCTTTTCTTCT-3′) (4th lane) and single stranded d(5′-AGAAGAAAAGA-3′) (5th lane) markers were used.

	A'_{BS}	A'_{SB}	A'_{AS}	A'_{TS}	A'_{CS}	A'_{BA}	A'_{AB}	A'_{BT}	A'_{BC}	E_{BS}	E_{AS}	E_{TS}	E_{CS}	E_{BA}	E_{BT}	E_{BC}
A'_{BS}	1.00	0.59	-0.09	0.12	-0.01	-0.21	-0.01	-0.08	-0.15	0.93	-0.08	0.10	-0.02	0.01	0.15	0.06
A'_{SB}	0.59	1.00	-0.09	-0.26	-0.01	0.36	-0.16	0.12	0.13	0.60	-0.10	-0.30	-0.02	-0.14	-0.44	-0.33
A'_{AS}	-0.09	-0.09	1.00	0.13	-0.03	0.08	0.21	-0.06	-0.04	-0.08	0.99	0.16	0.00	0.15	0.05	0.01
A'_{TS}	0.12	-0.26	0.13	1.00	0.06	-0.20	0.16	0.29	-0.05	0.08	0.14	0.94	0.07	0.15	0.54	0.26
A'_{CS}	-0.01	-0.01	-0.03	0.06	1.00	0.04	0.04	0.10	0.05	-0.03	-0.02	0.07	0.98	0.05	0.08	0.06
A'_{BA}	-0.21	0.36	0.08	-0.20	0.04	1.00	0.63	0.15	0.13	-0.30	0.08	-0.21	0.05	0.65	-0.38	-0.32
A'_{AB}	-0.01	-0.16	0.21	0.16	0.04	0.63	1.00	0.01	-0.11	-0.21	0.23	0.20	0.06	0.99	0.22	0.07
A'_{BT}	-0.08	0.12	-0.06	0.29	0.10	0.15	0.01	1.00	0.18	-0.04	-0.06	0.31	0.10	-0.02	0.56	0.01
A'_{BC}	-0.15	0.13	-0.04	-0.05	0.05	0.13	-0.11	0.18	1.00	-0.15	-0.03	-0.05	0.02	-0.11	-0.10	0.71
E_{BS}	0.93	0.60	-0.08	0.08	-0.03	-0.30	-0.21	-0.04	-0.15	1.00	-0.09	0.11	-0.03	-0.20	0.11	-0.01
E_{AS}	-0.08	-0.10	0.99	0.14	-0.02	0.08	0.23	-0.06	-0.03	-0.09	1.00	0.18	0.01	0.16	0.08	0.03
E_{TS}	0.10	-0.30	0.16	0.94	0.07	-0.21	0.20	0.31	-0.05	0.11	0.18	1.00	0.07	0.19	0.63	0.29
E_{CS}	-0.02	-0.02	0.00	0.07	0.98	0.05	0.06	0.10	0.02	-0.03	0.01	0.07	1.00	0.06	0.08	0.04
E_{BA}	0.01	-0.14	0.15	0.15	0.05	0.65	0.99	-0.02	-0.11	-0.20	0.16	0.19	0.06	1.00	0.18	0.05
E_{BT}	0.15	-0.44	0.05	0.54	0.08	-0.38	0.22	0.56	-0.10	0.11	0.08	0.63	0.08	0.18	1.00	0.41
E_{BC}	0.06	-0.33	0.01	0.26	0.06	-0.32	0.07	0.01	0.71	-0.01	0.03	0.29	0.04	0.05	0.41	1.00

Table S1. Correlation matrix corresponding to the model analysis of the DSC melting and cooling thermograms for concentration K^+ ions equal to 25 mM

Table S2. Correlation matrix corresponding to the model analysis of the DSC melting and cooling thermograms for concentration K+ ions equal to 35 mM.

	A'_{BS}	A'_{SB}	A'_{AS}	A'_{TS}	A'_{CS}	A'_{BA}	A'_{AB}	A'_{BT}	A'_{BC}	E_{BS}	E_{AS}	E_{TS}	E_{CS}	E_{BA}	E_{BT}	E_{BC}
A'_{BS}	1.00	0.55	-0.09	0.15	-0.01	-0.21	-0.01	-0.10	-0.15	0.95	-0.08	0.10	-0.02	0.01	0.16	0.06
A'_{SB}	0.55	1.00	-0.09	-0.26	-0.01	0.36	-0.16	0.12	0.15	0.55	-0.10	-0.30	-0.02	-0.14	-0.44	-0.33
A'_{AS}	-0.09	-0.09	1.00	0.13	-0.03	0.08	0.21	-0.06	-0.04	-0.08	0.99	0.16	0.00	0.15	0.05	0.01
A'_{TS}	0.15	-0.26	0.13	1.00	0.06	-0.20	0.16	0.29	-0.05	0.08	0.14	0.94	0.07	0.15	0.54	0.30
A'_{CS}	-0.01	-0.01	-0.03	0.06	1.00	0.05	0.05	0.10	0.05	-0.03	-0.02	0.07	0.99	0.05	0.10	0.06
A'_{BA}	-0.21	0.36	0.08	-0.20	0.05	1.00	0.63	0.15	0.13	-0.30	0.10	-0.21	0.05	0.65	-0.40	-0.32
A'_{AB}	-0.01	-0.16	0.21	0.16	0.05	0.63	1.00	0.01	-0.11	-0.25	0.23	0.20	0.06	0.99	0.22	0.07
A'_{BT}	-0.10	0.12	-0.06	0.29	0.10	0.15	0.01	1.00	0.18	-0.04	-0.06	0.31	0.08	-0.02	0.56	0.01
A'_{BC}	-0.15	0.15	-0.04	-0.05	0.05	0.13	-0.11	0.18	1.00	-0.15	-0.03	-0.05	0.02	-0.11	-0.10	0.75
E_{BS}	0.95	0.55	-0.08	0.08	-0.03	-0.30	-0.25	-0.04	-0.15	1.00	-0.09	0.11	-0.03	-0.20	0.11	-0.01
E_{AS}	-0.08	-0.10	0.99	0.14	-0.02	0.10	0.23	-0.06	-0.03	-0.09	1.00	0.18	0.01	0.16	0.08	0.03
E_{TS}	0.10	-0.30	0.16	0.94	0.07	-0.21	0.20	0.31	-0.05	0.11	0.18	1.00	0.07	0.19	0.63	0.29
E_{CS}	-0.02	-0.02	0.00	0.07	0.99	0.05	0.06	0.08	0.02	-0.03	0.01	0.07	1.00	0.06	0.10	0.04
E_{BA}	0.01	-0.14	0.15	0.15	0.05	0.65	0.99	-0.02	-0.11	-0.20	0.16	0.19	0.06	1.00	0.18	0.05
E_{BT}	0.16	-0.44	0.05	0.54	0.10	-0.40	0.22	0.56	-0.10	0.11	0.08	0.63	0.10	0.18	1.00	0.45
E_{BC}	0.06	-0.33	0.01	0.30	0.06	-0.32	0.07	0.01	0.75	-0.01	0.03	0.29	0.04	0.05	0.45	1.00

	A'_{BS}	A'_{SB}	A'_{AS}	A'_{TS}	A'_{CS}	A'_{BA}	A'_{AB}	A'_{BT}	A'_{BC}	E_{BS}	E_{AS}	E_{TS}	E_{CS}	E_{BA}	E_{BT}	E_{BC}
A'_{BS}	1.00	0.55	-0.09	0.15	-0.01	-0.21	-0.01	-0.10	-0.20	0.95	-0.05	0.10	-0.02	-0.01	0.16	0.06
A'_{SB}	0.55	1.00	-0.09	-0.26	-0.01	0.36	-0.16	0.10	0.15	0.55	-0.10	-0.30	-0.02	-0.14	-0.39	-0.33
A'_{AS}	-0.09	-0.09	1.00	0.13	-0.03	0.05	0.25	-0.06	-0.04	-0.08	0.95	0.16	0.00	0.15	0.05	0.01
A'_{TS}	0.15	-0.26	0.13	1.00	0.06	-0.20	0.16	0.29	-0.05	0.08	0.14	0.94	0.07	0.15	0.51	0.30
A'_{CS}	-0.01	-0.01	-0.03	0.06	1.00	0.05	0.05	0.10	0.05	-0.03	-0.02	0.07	0.99	0.05	0.10	0.06
A'_{BA}	-0.21	0.36	0.05	-0.20	0.05	1.00	0.63	0.15	0.13	-0.30	0.10	-0.21	0.05	0.65	-0.40	-0.32
A'_{AB}	-0.01	-0.16	0.25	0.16	0.05	0.63	1.00	0.01	-0.11	-0.25	0.25	0.20	0.06	0.99	0.22	0.07
A'_{BT}	-0.10	0.10	-0.06	0.29	0.10	0.15	0.01	1.00	0.18	-0.04	-0.06	0.31	0.08	0.02	0.56	0.01
A'_{BC}	-0.20	0.15	-0.04	-0.05	0.05	0.13	-0.11	0.18	1.00	-0.15	-0.03	-0.05	0.02	-0.11	-0.10	0.75
E_{BS}	0.95	0.55	-0.08	0.08	-0.03	-0.30	-0.25	-0.04	-0.15	1.00	-0.09	0.14	-0.03	-0.20	0.11	0.01
E_{AS}	-0.05	-0.10	0.95	0.14	-0.02	0.10	0.25	-0.06	-0.03	-0.09	1.00	0.18	0.01	0.16	0.08	0.03
E_{TS}	0.10	-0.30	0.16	0.94	0.07	-0.21	0.20	0.31	-0.05	0.14	0.18	1.00	0.07	0.19	0.63	0.29
E_{CS}	-0.02	-0.02	0.00	0.07	0.99	0.05	0.06	0.08	0.02	-0.03	0.01	0.07	1.00	0.06	0.10	0.05
E_{BA}	-0.01	-0.14	0.15	0.15	0.05	0.65	0.99	0.02	-0.11	-0.20	0.16	0.19	0.06	1.00	0.18	0.05
E_{BT}	0.16	-0.39	0.05	0.51	0.10	-0.40	0.22	0.56	-0.10	0.11	0.08	0.63	0.10	0.18	1.00	0.45
E_{BC}	0.06	-0.33	0.01	0.30	0.06	-0.32	0.07	0.01	0.75	0.01	0.03	0.29	0.05	0.05	0.45	1.00

Table S3. Correlation matrix corresponding to the model analysis of the DSC melting and cooling thermograms for concentration K+ ions equal to 50 mM.
